# Impact of post-challenge hyperglycemia on clinical outcomes in japanese patients with stable angina undergoing percutaneous coronary intervention

**DOI:** 10.1186/1475-2840-12-74

**Published:** 2013-05-07

**Authors:** Shoichi Kuramitsu, Hiroyoshi Yokoi, Takenori Domei, Akihiro Nomura, Hirotoshi Watanabe, Kyohei Yamaji, Yoshimitsu Soga, Takeshi Arita, Katsuhiro Kondo, Shinichi Shirai, Kenji Ando, Koyu Sakai, Masashi Iwabuchi, Hedeyuki Nosaka, Masakiyo Nobuyoshi

**Affiliations:** 1 Department of Cardiology, Kokura Memorial Hospital, 3-2-1 Asano, Kokurakita-ku, Kitakyushu 802-8555, Japan

**Keywords:** Coronary artery disease, Diabetes mellitus, Percutaneous coronary intervention, Post-challenge hyperglycemia, Stable angina

## Abstract

**Background:**

Post-challenge hyperglycemia (PH) is well-established as one of risk factors for coronary artery disease. However, it remains unclear whether PH affects clinical outcomes in patients with stable angina undergoing percutaneous coronary intervention (PCI).

**Methods:**

A total of 828 patients with stable angina undergoing PCI were retrospectively analyzed. Of these, 452 patients with previously diagnosed diabetes mellitus (DM) or fasting plasma glucose (PG) ≥126 mg/dl and HbA1c ≥6.5% were defined as known DM. The remaining 376 patients were divided into the two groups according to 2-h PG: PH (2-h PG ≥140 mg/dl, n=236) and normal glucose tolerance (NGT, 2-h PG <140 mg/dl, n=140). We assessed the rate of major adverse cardiovascular events (MACE), defined as a composite of cardiovascular death, myocardial infarction, stroke, and clinically-driven revascularization.

**Results:**

During the median follow-up of 4.3 years, the MACE rate was significantly higher in the DM and PH groups than the NGT group (39.3% vs. 20.7%, P <0.001; 31.4% vs. 20.7%, P=0.044, respectively). Compared with the NGT group, the cumulative incidence of revascularization was significantly higher in the DM group (35.1% vs. 18.5%, P <0.001) and tended to be higher in the PH group (27.1% vs. 18.5%, P=0.067). In the multivariate analysis, known DM (Hazard ratio [HR]: 2.16, 95% confidence interval (CI): 1.49-3.27, P < 0.001), PH (HR: 1.62, 95% CI: 1.07-2.53, P = 0.023), LDL-C >100 mg/dl (HR: 1.62, 95% CI: 1.26 to 2.10, P < 0.001), and previous stroke (HR: 1.47, 95% CI: 1.03-2.04, P = 0.034) were predictors of MACE.

**Conclusion:**

PH is associated with future cardiovascular events in patients with stable angina undergoing PCI.

## Introduction

Diabetes mellitus (DM) is an important public health problem. Its prevalence is variable and increases with age; in the USA 9.6% of the population aged over 20 years suffers from known DM [1]. DECODE (Diabetes Epidemiology: Collaborative Analysis of Diagnostic Criteria in Europe) study reported that the prevalence of DM and impaired glucose tolerance (IGT) in elderly individuals is 10 - 20% and 30 - 35%, respectively [2]. In Japan, the prevalence of glucose intolerance among the general population is 31.9%, which is increasing with time, possibly as a consequence of rapid changes in lifestyle by the Japanese population since the late 1980s [3,4].

Type 2 DM is associated with a 2- to 4-fold increased risk of cardiovascular events [5]. Several clinical trials have demonstrated that the post-challenge hyperglycemia (PH) is associated with adverse cardiovascular events [2,6-9]. Furthermore, a meta-analysis showed that hyperglycemia in the non-diabetic range was associated with the increased risk of fatal and non-fatal cardiovascular disease [10]. Macrovascular disease is a common diabetic complication and the leading cause of death among people with type 2 DM [11]. In particular, coronary artery disease (CAD) is one of major macrovascular complications in patients with type 2 DM.

In patients with CAD, the prevalence of DM is greater compared to that of general population [12-14]. Moreover, the prevalence of PH in patients undergoing angiography exceeds 60% and is associated with the angiographically determined extent of coronary artery disease [14]. However, the impact of PH on clinical outcomes in patients with stable angina undergoing percutaneous coronary intervention (PCI) has not been fully elucidated. Therefore, the aim of the present study was to evaluate whether newly diagnosed PH affects clinical outcomes in Japanese patients with stable angina undergoing PCI.

## Methods

### Study selection, procedure, and follow-up

A total of 1227 consecutive patients with stable angina underwent elective PCI with stent implantation at Kokura Memorial Hospital between October 2007 and March 2009. Of these, 277 patients were excluded because of (1) being older than 80 years; (2) a serum creatinine level ≥2.0 mg/dL; (3) type 1 DM; (4) concomitant diseases such as neoplasm, hepatic failure, or severe infection; (5) imcomplete revascularization at the time of hospital discharge; (6) complications associated with the procedure such as cerebral infarction and myocardial infarction; and (7) having acute coronary syndrome within 3-months prior to PCI. Finally, 950 patients were enrolled in this study. All interventions were performed using standard techniques. Predilatation, postdilatation, and stent selection were left to the operator’s discretion. After the procedure, all patients were advised to continue on aspirin (81 to 162 mg daily) for life unless there were contraindications. Either ticlopidine (200 mg daily) or clopidogrel (75 mg daily) was also prescribed for at least 1-month after bare-metal stent implantation, and 1 year after drug-eluting stent implantation. Follow-up data were retrospectively collected from a review of the hospital record or by telephone contacts with the patients, the family members, or the family physicians. This study was approved by the ethics committee of Kokura Memorial Hospital.

### Measurement and diagnosis of the glucometabolic status

The glucometabolic status of each patient before hospitalization was evaluated by medical questionnaire and outpatient records. Patients were classified as having known DM if they had a recorded history of DM or if they were on a diet or medical treatment for DM. In addition, patients with fasting plasma glucose (FPG) level ≥126 mg/dl and HbA1c ≥6.5% at the time of admission were also classified as having known DM. In patients without overt DM, a 75-g oral glucose tolerance test (OGTT) was performed at the time of discharge.

The remaining patients with FPG <126 mg/dl and HbA1c <6.5% were divided into the 2 groups according to 2-h plasma glucose (PG) classification adopted by American Diabetes Association criteria [15]. Patients with 2-h PG after the 75-g glucose load <140 mg/dl were classified as having a normal glucose tolerance (NGT). IGT was diagnosed in patients with 2-h PG between 140 and 199 mg/dl. Newly detected DM was diagnosed in patients with 2-h PG ≥200 mg/dl. According to the definition in a previous study [16], PH was defined as a composite of IGT and newly detected DM.

The value for HbA1c (%) was estimated as a National Glycohemoglobin Standardization Program equivalent value (%) calculated by the following formula: HbA1c (%)=HbA1c [value] +0.4, considering the relational expression of HbA1c (Japan Diabetes Society value, %) measured using the previous Japanese standard substance and measurement methods [17].

### Definitions of other coronary risk factors

Other coronary risk factors of each patient before hospitalization were evaluated by medical questionnaire and outpatient records. If the patients met the following criteria, these diseases were added to the baseline characteristics: hypertension, systemic arterial pressure >140 mmHg or diastolic arterial pressure >90 mmHg or treatment with anti-hypertensive agents; dyslipidemia, LDL cholesterol (LDL-C) >140 mg/dl or HDL cholesterol <40 mg/dl or triglyceride >150 mg/dl or treatment with any lipid lowering agents.

### Study endpoint and definitions

The study endpoint was the cumulative incidence of major adverse cardiovascular events (MACE), which was a composite of cardiovascular death, stroke, myocardial infarction (MI), clinically-driven revascularization. Cardiovascular death was defined as death from MI, stroke or sudden death without any obvious reasons. The diagnosis of acute MI (AMI) was established according to the universal definition of MI [18]. A clinically-driven revascularization was defined as treatment for recurrent angina in the presence of signs or symptom of myocardial ischemia, including target lesion revascularization (TLR), target vessel revascularization (TVR), non-target vessel revascularization (non-TVR), or coronary artery bypass graft (CABG). TLR was defined as repeat revascularization caused by a 50% stenosis within or within a 5-mm border proximal or distal stent. TVR was defined as any repeat PCI of any segment within the entire major coronary vessel that was proximal or distal to a target lesion, and the target lesion itself. Non-TVR was defined as any PCI of either of the major coronary arteries not including the target vessel.

### Statistical analysis

Data are presented as values and percentages, mean ± SD, or median (interquartile range). Analysis of normality of the continuous variable was performed with the Shapiro-Wilk test. Comparisons among the three groups (known DM, PH, and NGT) were examined using one-way analysis of variance (ANOVA) or Kruskal-Wallis test for continuous variables, and chi-square test or Fisher’s exact test for categorical variables, as appropriate. In the case of significant P values for ANOVA or Kruskal-Wallis test, the Tukey-Kramer test or Steel-Dwass test were used for multiple comparisons among groups, respectively. Comparisons for post-challenge 2-h PG between PH and NGT were made using the Mann–Whitney U test. The cumulative incidence of the MACE was estimated according to the Kaplan-Meier method. The log-rank test and hazard ratio were used to evaluate the differences between the incidence curves of the three groups. To identify independent predictors of the endpoints, Cox proportional hazard regression analysis was used. Univariate analysis was performed with 24 baseline characteristics and laboratory profiles. The insulin and OHA use were not included in univariate analysis because they were used only in patients with known DM. The following 8 variables with P <0.05 in the univariate analysis were tested for their multivariate predictive value: glucometabolic status (known DM, PH, and NGT), total cholesterol >220 mg/dl , LDL-C >100 mg/dl, previous stroke, FPG, and HbA1c.The final model was constructed using the 5 variables: glucometabolic status (known DM, PH, and NGT), previous stroke, and LDL-C >100 mg/dl selected by forward stepwise method, with entry and exit criteria test at the P=0.05 and P=0.10 levels, respectively. A 2-sided P value less than 0.05 was considered statistically significant. Statistical analysis was performed using JMP, version 10.0.2 (SAS Institute Inc., Cary, NC, USA) and SPSS, version 16 (SPSS Inc., Chicago, IL, USA)

## Results

### Study population

**Figure 1:**
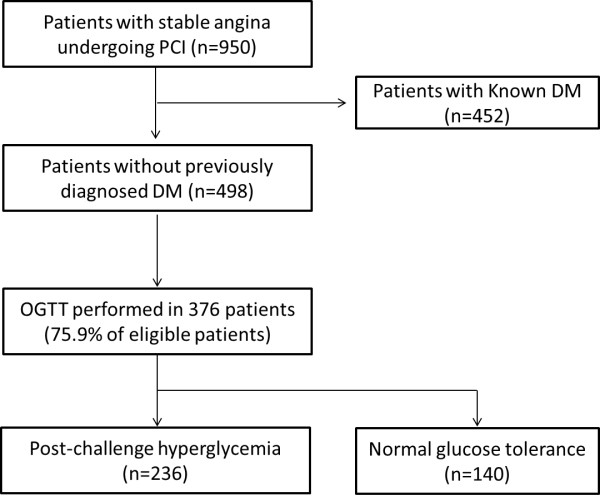
**Overview of patient enrollment.** DM = diabetes mellitus; OGTT = oral glucose tolerance test; PCI = percutaneous coronary intervention.

Of the 950 patients, the 75-g OGTT was not performed in 452 patients with known DM and another 120 patients without a previous diagnosis of DM. A 75-g OGTT was conducted on the remaining 378 patients (75.9% of the eligible population). According to the results of the 75-g OGTT, 236 (100 newly diagnosed DM and 136 IGT) and 140 patients were divided into PH and NGT patients, respectively. During the median follow-up period of 4.3 (interquartile range, 3.8 to 4.9) years, a total of 828 patients (452 DM, 236 PH, and 140 NGT) were analyzed (Figure 1).

### Baseline characteristics of the study population and glucometabolic status

The baseline characteristics of our study population are shown in Table 1. The median age was 69.0 years, and 78.8% of the patients were men. Most subjects had hypertension, followed by dyslipidemia. Compared with the NGT group, the body-mass index (BMI) and the prevalence of hypertension, previous PCI, oral hypoglycemic agents and insulin use were significantly higher, and the left ventricular ejection fraction was significantly lower in the DM group (P <0.05, respectively). Furthermore, the prevalence of male gender, BMI, and hypertension was significantly higher in the PH group compared with the NGT group (P <0.05, respectively). The glucometabolic states and lipid profile of the three groups are shown in Table 2. FPG and HbA1c levels were significantly higher in the DM group than those of the PH and NGT group (P <0.001, respectively). Compared with the NGT group, FPG, 2-h PG, and HbA1c levels were significantly higher in the PH group (p <0.001, respectively). In the lipid profile, HDL-cholesterol level was significantly lower in the PH groups compared with the DM and NGT groups (P <0.05, respectively).

**Table 1:** Baseline clinical characteristics of the 3 groups

	**DM (n=452)**	**PH (n=236)**	**NGT (n=140)**	**P value**
Age, years	70 (62–74)*	67 (59–72)	69 (62–74)	0.002
Male	347 (76.7)	203 (86.0)†‡	103 (73.5)	0.004
Hypertension	380 (84.0)	185 (78.3)	104 (74.2)	0.019
Dyslipidemia	295 (65.2)	164 (69.4)	101 (72.1)	0.24
Current smoking	119 (26.3)	74 (31.3)	34 (24.2)	0.24
Previous PCI	249 (55.0)*†	104 (44.0)	66 (47.1)	0.015
Previsou CABG	33 (7.2)	9 (3.8)	10 (7.1)	0.18
Previous MI	148 (32.7)	63 (26.6)	39 (27.8)	0.2
Previous CI	55 (12.1)	27 (11.4)	11 (7.8)	0.36
Multivessel disease	128 (28.3)	59 (25.1)	29 (20.7)	0.18
BMI, kg/m^2^	24.3 (22.1-26.4)†	24.7 (22.9-26.6)†	23.2 (21.0-25.7)	<0.001
LVEF, %	61.0 (50.0-68.0)*	64.0 (55.0-69.0)	64.0 (50.2-68.0)	0.017
eGFR, ml/min./1.73m^2^	64.9 (51.4-76.1)	66.2 (57.5-75.1)	65.6 (54.6-79.6)	0.17
Stent type				0.057
BMS	255 (56.4)	146 (61.8)	94 (67.1)	
DES	197 (43.6)	90 (38.2)	46 (32.9)	
Medication at discharge				
Aspirin	452 (100.0)	236 (100.0)	140 (100.0)	-
Thienopyridine	452 (100.0)	236 (100.0)	140 (100.0)	-
β-blocker	136 (30.0)	78 (33.0)	41 (29.2)	0.66
ACE-I	117 (25.8)	48 (20.3)	27 (19.2)	0.12
ARB	178 (39.3)	86 (36.0)	46 (32.8)	0.33
Statin	285 (63.0)	139 (58.9)	79 (56.4)	0.29
OHA	325 (71.9)	0 (0.0)	0 (0.0)	<0.001
Insulin	64 (14.1)	0 (0.0)	0 (0.0)	<0.001

Values are expressed as medians (interquartile range) or number and percentage. *P <0.05 vs. PH; †P <0.05 vs. NGT; and ‡P <0.05 vs. DM.

ACE-I, angiotensin-converting enzyme inhibitor; ARB, angiotensin receptor blocker; BMI, body-mass index; BMS, bare-metal stent; CABG, coronary artery bypass graft; CI, cerebral infarction; DES, drug-eluting stent; DM, diabetes mellitus; eGFR, Estimated glomerular filtrating ratio; LVEF, left ventricular ejection fraction; MI, myocardial infarction; NGT, normal glucose tolerance; OHA, oral hypoglycemia agent; PCI, percutaneous coronary intervention; PH, post-challenge hyperglycemia.

**Table 2:** Glucometabolic state and lipid profile of the 3 groups

	**DM (n=452)**	**PH (n=236)**	**NGT (n=140)**	**P value**
Glucometabolic state				
FPG, mg/dl	140.0 (111.2-194.0)*†	94.0 (88.0-101.7)†	86.0 (82.2-91.0)	<0.001
2-h PG, mg/dl	-	189.5 (161.0-224.2)†	116.0 (97.0-128.0)	<0.001
HbA1c, %	7.3 (6.8-8.3)*†	6.0 (5.8-6.3)†	5.8 (5.6-6.0)	<0.001
Lipid profile, mg/dl				
Total choresterol	181 (156–207.7)	176 (155.2-196.7)	177.5 (158.2-203.7)	0.37
Triglyceride	131.5 (94–194)	132 (101.2-178.7)	117 (91.5-162)	0.12
HDL-C	47 (40–55)	44 (37–51)†‡	47.5 (39–57)	0.001
LDL-C	104 (85–126.7)	106.5 (88.2-126)	105.5 (88–125.7)	0.64

Values are expressed as medians (interquartile range). *P <0.05 vs. PH; †P <0.05 vs. NGT; and ‡P <0.05 vs. DM.

FPG, fasting plasma glucose; HbA1c, hemoglobin A1c; HDL-C, high-density lipoprotein cholesterol; LDL-C, low-density lipoprotein cholesterol; PG, plasma glucose. Other abbreviations as in Table 1.

### Post-challenge hyperglycemia, known DM, and MACE

**Figure 2:**
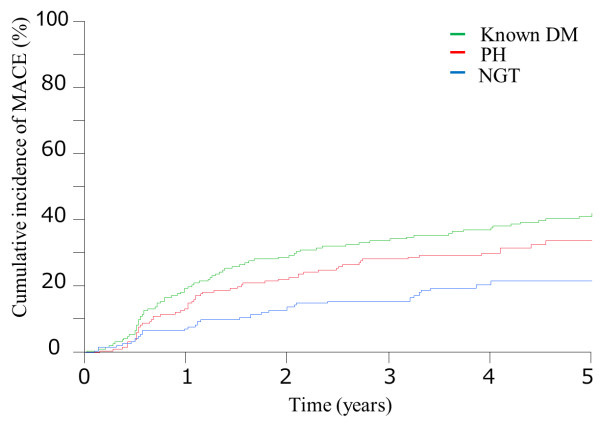
**Kaplan-Meier plot of the cumulative incidence of the study endpoint between the three groups.** The study endpoint was defined as cardiovascular death, myocardial infarction, stroke, and clinically-driven revascularization. Known DM = diabetes mellitus (green line); NGT = normal glucose tolerance (blue line); PH = post-challenge hyperglycemia **(red line)**.

Clinical events in the three groups are summarized in Table 3. As shown in Figure 2, the cumulative incidence of MACE was significantly higher in the DM and PH groups than in the NGT group (39.3% vs. 20.7%, P <0.001; 31.4% vs. 20.7%, P=0.044, respectively), and there was trend toward the higher MACE rate in the DM group compared to the PH group (39.3% vs. 30.9%, P=0.056). Compared with the NGT group, the cumulative incidence of revascularization was significantly higher in the DM group (35.1% vs. 18.5%, P <0.001) and tended to be higher in the PH group (27.1% vs. 18.5%, P=0.067).

**Table 3:** Cumulative incidence of clinical events during the follow-up period

	**DM (n=452)**	**PH (n=236)**	**NGT (n=140)**	**P value**
MACE	178 (39.3)*†	74 (31.4)†	29 (20.7)	<0.001
All-cause Death	37 (8.1)*†	8 (3.3)	3 (2.1)	0.006
Non-cardiovascular	21 (4.6)	4 (1.6)	3 (2.1)	0.08
Cardiovascular	16 (3.5)	4 (1.6)	0 (0.0)	0.041
Stroke	20 (4.4)	8 (3.3)	3 (2.1)	0.5
MI	12 (2.6)	6 (2.5)	2 (1.4)	0.7
Revascularization	159 (35.1)*†	64 (27.1)	26 (18.5)	<0.001
PCI	157 (34.7)*†	63 (26.6)	26 (18.5)	<0.001
TLR	47 (10.3)†	13 (5.5)	4 (2.8)	0.005
TVR	80 (17.6)*†	27 (11.4)	10 (7.1)	0.002
Non-TVR	101 (22.3)	42 (17.8)	19 (13.5)	0.055
CABG	12 (2.6)	3 (1.2)	0 (0.0)	0.09

Values are expressed as number and percentage. *P <0.05 vs. PH; †P <0.05 vs. NGT. TLR, target lesion revascularization; TVR, target vessel revascularization. Other abbreviations as in Table 1.

In the Cox regression analysis, known DM (Hazard ratio [HR]: 2.16, 95% confidence interval (CI): 1.49-3.27, P < 0.001), PH (HR: 1.62, 95% CI: 1.07-2.53, P = 0.023), LDL-C >100 mg/dl (HR: 1.62, 95% CI: 1.26 to 2.10, P < 0.001), and previous stroke (HR: 1.47, 95% CI: 1.03-2.04, P = 0.034) were predictors of MACE in patients with stable angina undergoing PCI (Table 4).

**Table 4:** Predictors of major adverse cardiovascular events

	**Adjusted HR**	**95% CI**	**P value**
Glucometabolic state			
Normal glucose tolerance	1.00		
Post-challenge hyperglycemia	1.59	1.04-2.48	0.029
Known DM	2.12	1.46-3.21	<0.001
LDL-cholesterol >100 mg/dl	1.62	1.26-2.10	<0.001
Previous stroke	1.46	1.03-2.02	0.034

CI=confidence interval; HR=hazard ratio. Other abbreviations as in Table 1.

### Impaired glucose tolerance, newly detected DM, and MACE

**Figure 3:**
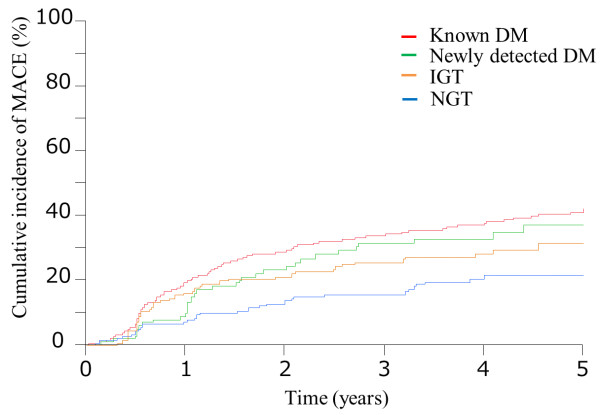
**Kaplan-Meier plot of the cumulative incidence of the study endpoint between the 4 groups.** Known DM = diabetes mellitus (red line); Newly diagnosed DM = diabetes mellitus (green line); IGT = impaired glucose tolerance (brown line); NGT = normal glucose tolerance (blue line).

Figure 3 shows the results of a secondary analysis addressing the cumulative incidence of MACE in the categories of newly detected DM, IGT, NGT, and known DM. Compared with the NGT group, there was trend toward the higher MACE rate in the newly detected DM group compared to the NGT group (34.0% vs. 20.7%, P=0.075), whereas no significant difference in MACE rate was found between patients with IGT and NGT (P=0.27). There was no significant difference in the rate of revascularization between the newly detected DM, IGT, and NGT groups (Additional file 1: Table S1).

After adjustment for known DM, newly detected DM, IGT, NGT, LDL-C >100 mg/dl, and previous stroke in Cox regression analysis, adjusted HR were 1.75 (95% CI: 1.07-2.89, P=0.027), 1.53 (95% CI: 0.95-2.48, P=0.08), and 2.16 (95% CI: 1.49-3.27, P <0.001) for patients with newly detected DM, IGT, and known DM, respectively, when compared with the NGT subjects.

## Discussion

The main findings of the present study are: (1) our study was the analysis of a homogeneous group of patients with stable angina undergoing PCI; (2) PH was associated with future cardiovascular events in patients with stable angina undergoing PCI, which is mainly due to the need for repeated revascularization; (3) The prevalence of glucometabolic abnormalities in our study was very high (72.4%). Of these, 34.3% were classified as PH, which was diagnosed using OGTT; (4) Known DM, PH, LDL-C >100 mg/dl, and previous stroke were independent predictors of MACE in patients with stable angina undergoing PCI.

Several studies have reported the impact of different glucose abnormalities on clinical outcomes in patients with cardiovascular disease [16,19-25]. However, most of the studies were performed on heterogeneous groups of patients, i.e., those with stable and unstable angina, myocardial infarction, or heart failure. Furthermore, these patients were also treated with multiple modalities, and only in a few studies were all subjects treated with PCI. Therefore, the main advantage of the present study was the analysis of a homogeneous group of patients with stable angina undergoing PCI.

The present study showed that PH was associated with future cardiovascular events in patients with stable angina undergoing PCI, which is mainly due to the need for repeated revascularization. Previous studies have shown that PH and hyperinsulinemia disrupt normal endothelial function and accelerate the formation of atherosclerotic plaques, leading to plaque rupture and subsequent thrombus formation [26-29]. Furthermore, PH and hyperinsulinemia have been shown to be predictive of restenosis after BMS implantation in nondiabetic patients [30-32]. These findings are consistent with our current observations. The data available to date, however, are insufficient to support the lowering of PH as a primary target to reduce the risk of macrovascular events in patients with stable angina. Recently, the DIANA (Diabetes and diffuse coronary Narrowing) study reported that improvement in glycemic status at 1 year was associated with less atheroma progression in Japanese early-stage DM patients with CAD, regardless of the treatment, including life-style intervention, voglibose or nateglinide treatment [33]. Although this study could not assess the impact of improving glycemic status on MACE, it suggests the need for intensive management of glycemic abnormalities in early-stage DM. Furthermore, the benefits of early intensive therapy to control blood glucose levels in patients with newly diagnosed DM were sustained for up to 10 years after cessation of intensive therapy [34]. Therefore, PH might be considered a treatment target in patients with stable angina undergoing PCI.

The prevalence of glucometabolic abnormalities in our study was very high (72.4%). Of these, 34.3% were classified as PH, which was diagnosed using OGTT. This is concordant with the results from previous studies [9,12-14,16,35]. While OGTT is an indispensable tool to detect glucometabolic abnormalities [36,37], FPG is still often used for the diagnosis in daily practice. However, as shown in DECODA (Diabetes Epidemiology: Collaborative Analysis of Diagnostic Criteria in Asia) study [9], it would be inappropriate to use the FPG criteria alone for screening diabetes in Asian populations. Furthermore, current guidelines recommend that 75-g OGTT should be considered in high-risk patients, i.e., those with cardiovascular disease and an Hb1Ac level of 5.7 to 6.4% [38,39]. Therefore, these facts suggest that it mandatory to perform OGTT in patients with stable angina undergoing PCI, unless a glucometabolic abnormality has been already established.

It has been reported that established DM deteriorates the prognosis of patients with stable angina. However, the impact of prediabetes (IGT and impaired fasting glucose) on the clinical outcomes in patients with CAD remains controversial. In our study, newly detected DM affected clinical outcome, whereas IGT did not. In contrast, Sourij *et al.*[16] reported that vascular risk was already significantly increased in angiographied coronary patients with PH in the IGT range. Although this report demonstrated an issue similar to our study, there were a few differences between the two studies. Most importantly, significant stenoses were found in 57.4-68.0% of subjects in this study, whereas all subjects underwent PCI and 26% had multivessel coronary disease. While it remains unclear how the patients were treated in this study, the difference of the study population between the two studies might affect the results. To elucidate the impact of prediabetes on clinical outcome in CAD patients, further larger investigations will be needed.

Clinical trials using statins to lower LDL-C have demonstrated reductions in cardiovascular events and atheroma progression [40-42]. Interestingly, Daida *et al.* reported that plaque regression was less pronounced in patients with high HbA1c levels compared with those with low levels [43]. Thus, tight glucose control during statin therapy may enhance plaque regression in patients with stable angina. Furthermore, Bayturan *et al.*[44] reported that the presence of DM and increased systolic blood pressure were independently associated with plaque progression in patients with LDL-C ≤70 mg/dl. These findings support the concept that coronary atherosclerosis is a multifactorial process that is likely to respond to global risk modification. In the present study, LDL-C >100 mg/dl was an independent predictor of MACE in patients with stable angina undergoing PCI, whereas statins were used in approximately 60% of the subjects. Furthermore, ACE-I and ARB were also used in only 60% of the subjects. This may be due to our inadequate awareness regarding lipid lowering therapy and antihypertensive agent for patients with stable angina in the present study. Therefore, we should reaffirm the importance of multifactorial intervention for coronary risk factors to prevent future cardiovascular events in patient with stable angina and glucometabolic abnormalities.

### Study limitations

There were several limitations in the present study. First, the present study was a retrospective cohort study at a single center. In addition, a quarter of eligible patients did not have OGTT. Although the baseline characteristics between patients with and without OGTT were similar (Additional file 1: Table S2), selection bias may exist and may have affected the conclusion. Second, 75-g OGTT was performed 2 days after PCI. Ideally, it should have been done prior to the PCI, but this was impossible due to logistic reasons. However, a meta-analysis has shown that it is reasonable to screen patients hospitalized for ACS for previously undiagnosed DM using an OGTT [45]. Moreover, all subjects in the present study had stable angina. Therefore, the result of OGTT in the present study was thought to be acceptable. Third, we excluded the patients with >80 years old and serum creatinine >2.0 mg/dl in the present study because we had performed OCTT based on a previous study [19]. Therefore, further study will be needed to assess the impact of PH on future cardiovascular events in clinical practice. Fourth, we could not assess the impact of impaired fasting glucose (IFG; FPG 110–125 mg/dl and 2-h PG <140 mg/dl) on clinical outcomes in the present study because patients with IFG were observed in only 2 patients. Fifth, we could not follow the changes in therapeutic agents during the follow-up. Therefore, these findings may have biased the conclusion. Finally, we could not identify whether early intervention for PH results in good clinical outcomes in the present study. Further study is needed to identify the optimal therapies for patients with stable angina and PH.

## Conclusions

PH is associated with future cardiovascular events in patients with stable angina undergoing PCI. Our results suggest that PH might be considered as a target of treatment to prevent future cardiovascular events in patients with stable angina undergoing PCI.

## Abbreviations

AMI: Acute myocardial infarction; ANOVA: One-way analysis of variance; CABG: Coronary artery bypass graft; CAD: Coronary artery disease; COSMOS: The Coronary Atherosclerosis Study Measuring Effects of Rosuvastatin Using Intravascular Ultrasound in Japanese Subjects; DECODA: Diabetes Epidemiology, Collaborative Analysis of Diagnostic Criteria in Asia; DECODE: Diabetes Epidemiology, Collaborative Analysis of Diagnostic Criteria in Europe; DIANA: Diabetes and diffuse coronary Narrowing; DM: Diabetes mellitus; eGFR: Estimated glomerular filtrating ratio; FPG: Fasting plasma glucose; GAMI: Glucose Tolerance in Patients with AMI; IGT: Impaired glucose tolerance; IFG: Impaired fasting glucose; LDL-C: LDL cholesterol; MACE: Major adverse cardiovascular events; MI: Myocardial infarction; NGT: Normal glucose tolerance; OGTT: Oral glucose tolerance test; PCI: Percutaneous coronary intervention; PG: Plasma glucose; PH: Post-challenge hyperglycemia; TLR: Target lesion revascularization; TVR: Target vessel revascularization

## Competing interests

The authors declare that they have no competing interests.

## Authors’ contributions

SK drafted the manuscript; HY, KK, SS, KA and KS conceived of and designed the study; KY, YS and TA contributed to the analysis of the data; TD, AN and AN collected data; and MI, HN and MN coordinated and managed the study. All authors read and approved the final manuscript.

## Supplementary Material

Additional file 1: Table S1 Cumulataive incidence of clinical events in patients with known DM, newly-detected DM, IGT, and NGT. **Table S2.** Baseline Characteristics in patients with and without OGTT. Click here for file
